# Loneliness in pregnant and postpartum people and parents of children aged 5 years or younger: a scoping review protocol

**DOI:** 10.1186/s13643-020-01469-5

**Published:** 2020-09-14

**Authors:** Jacqueline Kent-Marvick, Sara Simonsen, Ryoko Pentecost, Mary M. McFarland

**Affiliations:** 1 College of Nursing, University of Utah, 10 2000 E, Salt Lake City, UT 84112 USA; 2 Eccles Health Sciences Library, University of Utah, 10 N 1900 E, Salt Lake City, UT 84112 USA

**Keywords:** Loneliness, Maternal loneliness, Parental loneliness, Loneliness in parenthood, Loneliness in pregnancy, Scoping review, Scoping review protocol

## Abstract

**Background:**

The experience of loneliness during pregnancy and in new parenthood has not been targeted and developed as a program of research, despite evidence indicating that the incidence of loneliness is highest in those aged 16 to 24 and that loneliness rises during transitional periods. The scarcity of parenthood-loneliness inquiries leaves a gap in our understanding of new parenthood and its effects on the health and well-being of parents and their children. Here, a scoping review protocol will be presented to address this gap. The objective of this study will be to summarize the current knowledge of loneliness experienced during pregnancy and by parents during the postpartum period through the first 5 years of the child’s life.

**Methods:**

A scoping review protocol was designed following Arksey and O’Malley’s framework. We will include all types of literature in English, including all study designs, reviews, opinion articles, dissertations, reports, books, and grey literature. To be considered for inclusion, sources should focus on loneliness in pregnant persons, postpartum people, and parents of children 5 years or younger. We will search the following electronic databases (from inception onwards): MEDLINE, EMBASE, CINAHL Complete, Cochrane Library, PsycINFO, Dissertations & Theses Global, Sociological Abstracts, Scopus, and Web of Science. Grey literature will be identified searching the British governmental website gov.uk, the Jo Cox Commission on Loneliness, the Campaign to End Loneliness, and the British Red Cross’s Action on Loneliness websites. Two reviewers, working independently of each other, will screen the titles and abstracts of the articles returned by the searches, then screen the selected full-text articles, and extract data. A third reviewer will cast the deciding vote in case no consensus is reached. Results will be given in the narrative form, mapped, and illustrated.

**Discussion:**

This scoping review will capture the state of the current literature on loneliness in pregnancy and new parenthood. Results will be published in a peer-reviewed journal. We anticipate that the study will identify gaps and make recommendations for future areas of study and related interventions. The protocol is available on Open Science Framework at DOI 10.17605/OSF.IO/BFVPZ.

## Background

Despite extensive loneliness research primarily addressing the elderly and/or other high-risk groups across the lifespan, parents and pregnant people, as a group, remain an underrepresented population within loneliness studies [1], and few studies of new parents have made the examination of loneliness a primary aim (see exceptions [2–18]). There is a gap, therefore, in our understanding of the psychosocial needs and health risks of loneliness in pregnant persons and new parents.

Loneliness is the negative feeling associated with the appraisal of one’s social network as somehow deficient, as, for example, in the quantity of social contacts and/or quality of desired types of relationships [19]. This subjective experience is fundamentally different from the objective condition of being socially isolated [19]. Because new parenthood is a time of transition, particularly germane to new parenthood is transitional loneliness, which is loneliness experienced during crises and developmental changes throughout the lifespan [20]. Examples of transitional loneliness include the death of a close relation or the transition to college life.

The many loneliness and social isolation studies across the lifespan have linked the condition of perceived deficiencies in personal networks (e.g., friends, family members, co-workers, etc.) to far-reaching physical and mental effects [21, 22]. These morbidity factors across age groups include an increased mortality risk comparable to smoking up to 15 cigarettes per day [23], depression, increased systolic blood pressure, impaired sleep [24], and increased rate of physiological deterioration [25], to name a few.

There is a need to understand the breadth of the literature and to recognize the gap in perinatal loneliness studies because the prevalence of loneliness in new parenthood has been shown to be greater than in the general population. In a large-scale 2018 survey of US residents, 54% of the 10,000 US respondents reported feeling lonely [26]. In the survey’s 2019 follow-up that number had climbed to 61% [26]. There is, however, limited data about the prevalence of loneliness among new parents. A 2018 survey revealed that within this population, 32% always or often felt lonely, and 82% felt lonely at least some of the time [27]. Moreover, persons aged 16 to 24 most frequently reported loneliness [28]. These two important findings suggest that young parents might be at particular risk for loneliness during the transition to parenthood. To the author’s knowledge, however, the incidence of loneliness among new parents within this age group has never been specifically considered.

Because loneliness in this population has not been developed through a rigorous program of research, and is in a relatively amorphous state, little is known about the overall effects of loneliness during pregnancy and in new parenthood. The approach of a scoping review will be adopted in this case in order to map the literature to date and help guide future research and intervention development.

### Aims and review questions

This scoping review will aim to capture the state of the current literature on loneliness in pregnancy and new parenthood in order to identify gaps and make recommendations for future areas of study and related interventions.

Our primary research question will be: What is the research or evidence about loneliness in pregnancy and parents of children aged 5 years or younger? Secondary research questions will be: What are the targeted populations (e.g., parental ethnicity, socioeconomic situation, age, etc.) in the research done to date on the topic of loneliness during pregnancy and in new parents? What methodologies have been used to research the topic, and how has loneliness been measured and defined in this population? And what, if any, questions that help to define the nature of parental loneliness have already been answered (e.g., how common is loneliness within this group? Are there differences between stratified groups within this population? What are the associated factors? What factors are protective? How does loneliness affect bonding? Does it affect the child’s development? etc.)?

## Methods

The review protocol has been registered with the Open Science Framework database (registration number: DOI 10.17605/OSF.IO/BFVPZ) and is being reported in accordance with the reporting guidance provided in the Preferred Reporting Items for Systematic Reviews and Meta-Analyses Protocols (PRISMA-P) statement [29–31] (See checklist in Additional file 1). In accordance with scoping review procedure, we will conduct our scoping review as outlined by Arksey [32] and expanded by Peters [33] and The Joanna Briggs Institute [34] using Arksey’s five stages: (1) identifying the research question, (2) identifying relevant studies, (3) selecting studies, (4) charting the data, and (5) collating, summarizing, and reporting the results. We will follow the PRISMA reporting guidelines for scoping reviews to ensure transparency and reproducibility [31]. The evidence will be conducted in a transparent, replicable format in order to identify, characterize, summarize and map existing literature on loneliness in pregnancy, the postpartum period, and new parenthood during the 5 years following the child’s arrival and up to the age of 5.

### Eligibility criteria

The Population-Concept-Context (PCC) framework will be used to align the study selection with the research question [34]. To be included in the review, sources of evidence will need to include people who are pregnant, are parents in the postpartum period, or have children under the age of 5. The decision was made to include parents of children under the age of 5, as parenting demands of young children are generally greater in children aged 1 through 5, and this represents a time of rapid transition of life responsibilities and parenting demands. The phenomenon of interest for this scoping review will be the concept of loneliness—the negative feeling associated with the appraisal of one’s social network as somehow deficient, such as in the quantity of social contacts and/or quality of desired types of relationships [19]. This scoping review will consider any research relating to loneliness experienced during pregnancy, the postpartum period, or during the 5 years after the child’s birth, as the aim will be to map the current literature on this topic. We will, however, exclude studies that are primarily interested in loneliness experienced by children of any age, as well as articles where no English-language translation is available.

### Information sources and search strategy

The primary source of literature will be a structured search of multiple electronic databases (from inception onwards): MEDLINE (Ovid), EMBASE (embase.com), SCOPUS (scopus.com), Cochrane Library including CENTRAL (Wiley), CINAHL (Ebscohost), PsycINFO (Ebscohost), Dissertations & Theses Global (ProQuest) and Sociological Abstracts (ProQuest) and the Web of Science Core Collection (Clarivate). The secondary source of potentially relevant material will be a search of the grey literature, including the British governmental website, gov.uk, the Jo Cox Commission on Loneliness, the Campaign to End Loneliness, and the British Red Cross’s Action on Loneliness websites. Additional sites may be identified from these sources. The references of included documents will be used to identify any additional sources of evidence. Efforts will be made to contact authors of completed studies, ongoing studies, and in-press or unpublished literature for additional information or relevant material. An information specialist will develop the search strategy for our primary database, MEDLINE (Ovid), then translate for the databases. The search strategies will be peer-reviewed by library colleagues using the Peer Review of Electronic Search Strategies (PRESS) checklist [35]. Strategies will use database subject terms and keywords for loneliness and pregnancy or parenting or parents or mothers or fathers (expecting or child-rearing). A draft search strategy for MEDLINE (Ovid) is provided in Additional file 2.

### Selection of sources of evidence

Two reviewers will screen all articles returned by the searches independently. First, titles and abstracts of articles returned from initial searches will be screened based on the eligibility criteria outlined above. Second, full-text articles will be examined in detail and screened for eligibility. Third, references of all considered articles will be hand-searched to identify any relevant publications missed in the search strategy by two reviewers independently. Any disagreement between reviewers will be resolved by discussion to meet a consensus, and a third reviewer will be available to cast a deciding vote in the case that no consensus can be reached between the two reviewers. EndNote version X9 (Clarivate Analytics) will be used to manage citations and remove duplicates. Covidence (Veritas Health Innovation), an online systematic platform, will be used to screen and review search results [36].

### Data extraction

REDCap electronic data capture tools hosted at the University of Utah will be used to chart extracted data from our selected articles [37, 38]. Data extraction forms will be piloted initially on a small number of included articles. Each of the included articles will be charted by two reviewers independently. Potential conflicts will be resolved through discussion, and a third reviewer may be included to assist with determining results in the case that conflict is difficult to settle. Authors of primary publications will be contacted for data clarifications or missing outcome data, as necessary. Information of interest will include the following: author(s); year of publication; source origin/country of origin; aims/purpose; study population and sample size; methodology; intervention type and comparator; duration of the intervention; method of measuring outcomes; outcomes/important results; missing data; type of loneliness identified; definition of loneliness used; factors associated with loneliness; factors that protect against loneliness; effects of loneliness on pregnancy outcomes and/or on the child; prevalence of loneliness.

### Quality appraisal

In compliance with scoping review methodology [32–34], no quality assessment of selected articles will be performed, as our goal is to rapidly map the literature and identify gaps in knowledge.

### Data synthesis

The results of the scoping review will be synthesized into a narrative summary in relation to the study objective, research questions, and eligibility criteria (PCC framework). Results will be thematically sorted and arranged according to these criteria. Quantitative data will be summarized as numerical counts and frequencies. We will use tables and graphs to summarize articles, their characteristics, definitions of loneliness, associated factors, and other main findings, based on data extraction criteria. Suggestions for future research based on the study findings will also be summarized.

## Discussion

The review will aim to reveal the extent and types of study on loneliness in pregnancy and new parents by mapping the literature to date, including the methodologies used to research the topic, the means of measuring and defining loneliness in pregnancy and the new-parent population, and those questions, if any, which have already been answered concerning the definition and nature of loneliness in pregnant women and new parents. We will therefore seek to capture the state of the current literature on loneliness in pregnancy and new parenthood in order to identify gaps and make recommendations for future areas of study and related interventions.

To reduce bias, we will search for a range of databases and grey literature. Potential limitations of this scoping review may include the determination of inclusion or exclusion through the reviewers’ subjective measurement of the degree to which loneliness is featured in the selected articles. For example, loneliness may be briefly discussed in a qualitative study, but not be a prominent finding or emerge as a theme from the study, and this might result in the article’s exclusion from the scoping review. Any amendments made to this protocol when conducting the study will be outlined and reported in the final manuscript.

Following the completion of data extraction, results will be disseminated through publication in a peer-reviewed journal. An abstract will be submitted to the Western Institute of Nursing for their virtual 2021 conference.

## Supplementary information

**Additional file 1.** Preferred Reporting Items for Systematic reviews and Meta-Analyses extension for Scoping Reviews (PRISMA-ScR) Checklist.

**Additional file 2.** Database: Ovid MEDLINE(R) and Epub Ahead of Print, In-Process & Other Non-Indexed Citations, Daily and Versions(R) <1946 to January 10, 2020> Search Strategy.

## Data Availability

Not applicable.
