# Supplementary material for: Loneliness in pregnant and postpartum people and parents of children aged 5 years or younger: a scoping review protocol
Source: Syst Rev. 2020 Sep 14;9:213. doi: 10.1186/s13643-020-01469-5 (PMC7491158; doi:10.1186/s13643-020-01469-5)
Supplement: Supplementary file 2 — Additional file 2. Database: Ovid MEDLINE(R) and Epub Ahead of Print, In-Process & Other Non-Indexed Citations, Daily and Versions(R) <1946 to January 10, 2020> Search Strategy. [file 13643_2020_1469_MOESM2_ESM.docx]

Medline Final Jan 13, 2020 ScR 2019 PHR-2670

Database: Ovid MEDLINE(R) and Epub Ahead of Print, In-Process & Other Non-Indexed Citations, Daily and Versions(R) <1946 to January 10, 2020> Search Strategy:

--------------------------------------------------------------------------------

1 Loneliness/ (3465)

2 (loneliness or lonely).ti,ab,kf,kw,hw. (7600)

3 or/1-2 [ loneliness set ] (7600)

4 exp Pregnancy/ (879043)

5 (pregnant or pregnanc* or "child bearing" or childbearing or gestation or gravidity or antetnatal or "ante natal" or prenatal or pre-natal).ti,ab,hw,kw. (1034757)

6 or/4-5 [ pregnant set ] (1048571)

7 exp Pregnancy Complications/ (418105)

8 (pregnanc* adj2 complicat*).ti,ab,hw,kw. (171497)

9 or/7-8 [ pregnancy complications set ] (423931)

10 Parturition/ or peripartum period/ or postpartum period/ (34079)

11 (parturition* or birth? or childbirth? or peripartum or postpartum or perinatal or peri-natal or postnatal or post-natal or puerperium).ti,ab,hw,kw. (538173)

12 or/10-11 [ parturition set ] (538173)

13 Depression, Postpartum/ (5268)

14 ((depression or depressive) adj2 (postpartum or post-partum or postnatal or post-natal or "post natal" or antetnatal or "ante natal" or prenatal or pre-natal)).ti,ab,hw,kw. (8349)

15 or/13-14 [postpartum/prenatal depression set] (8349)

16 Parenting/ (16059)

17 (parenting or childrearing or "child rearing").ti,ab,hw,kf,kw. (31903)

18 or/16-17 [ Parenting set] (31903)

19 parents/ or fathers/ or mothers/ or single parent/ or surrogate mothers/ (108790)

20 (parent or parental or parents or mother or mothers or father or fathers).ti,ab,hw,kw. (571992)

21 or/19-20 [ parents set] (571992)

22 parent-child relations/ or father-child relations/ or mother-child relations/ or maternal-fetal relations/ (55369)

23 ((parent-child or father-child or mother-child or maternal-fetal) adj2 relation?).ti,ab,hw,kw. (55510)

24 or/22-23 [ parent-child relations set ] (55510)

25 maternal behavior/ or paternal behavior/ (12161)

26 ((maternal or paternal) adj2 behavio?r*).ti,ab,hw,kw. (14311)

27 or/25-26 [ maternal/paternal behavior set ] (14311)

28 ("28795604" or "17451030" or "26211398" or "31349835").ui. [ exemplars] (4)

29 exp animals/ not humans.sh. [Animals set] (4663411)

30 and/3,6 [ Lonelieness + pregnancy set ] (166)

31 and/3,9 [ Loneliness + pregnancy complications set] (49)

32 and/3,12 [ Lonelliness + parturition set ] (170)

33 and/3,15 [ Loneliness + postpartum depression set ] (24)

34 and/3,18 [ Loneliness + parenting set] (118)

35 and/3,21 [ Loneliness + parents set ] (796)

36 and/3,24 [Loneliess + parent-child relations set] (203)

37 and/3,27 [ Loneliness + maternal/paternal behavior set ] (15)

38 (or/30-37) not 29 [Final set] (963)

39 or/28,38 [Final set finds exemplars] (963)

40 remove duplicates from 39 (961)
